# Prevalence of Overweight and Obesity and Their Associated Factors Among Adolescent Children in an Urban School in Tamil Nadu

**DOI:** 10.7759/cureus.103933

**Published:** 2026-02-19

**Authors:** Asher Edward Prem Kumar, Kannan Lakshminarayanan

**Affiliations:** 1 Community Medicine, Sri Ramachandra Institute of Higher Education and Research, Chennai, IND

**Keywords:** adolescents, body mass index, fast food consumption, obesity, overweight, public health, screen time, urban health, well-being

## Abstract

Background

Overweight and obesity among adolescents have emerged as major public health concerns, particularly in urban India, due to rapid lifestyle transitions, unhealthy dietary practices, and increased sedentary behavior. Adolescence is a critical period during which excess weight often tracks into adulthood, increasing the risk of non-communicable diseases. However, city-specific data from metropolitan areas such as Chennai remain limited.

Methods

A school-based cross-sectional study was conducted from January 2025 to April 2025 among 222 adolescents aged 13-18 years in Chennai, Tamil Nadu. Data on socio-demographic characteristics and lifestyle behaviors were collected using a pre-tested structured questionnaire. Anthropometric measurements were recorded using standard procedures, and body mass index (BMI) was calculated and classified according to WHO BMI-for-age Z-scores (5-19 years). Associations were assessed using chi-square tests and odds ratios with 95% confidence intervals, followed by multivariate logistic regression to identify independent predictors.

Results

A total of 222 adolescents were included in the study.* *The overall prevalence of overweight and obesity was 21.7%, with 7.7% classified as overweight and 14.0% as obese. Female adolescents had significantly higher odds of being overweight or obese compared to male adolescents (OR = 6.11; 95% CI: 2.40-15.60). Excess daily screen time (>2 hours/day), frequent fast-food consumption (≥3 times/week), and late dinner timing (after 9 PM) were significantly associated with excess body weight on bivariate analysis. On multivariate analysis, female gender (AOR = 6.52), increased screen exposure (AOR = 2.46), and frequent fast-food intake (AOR = 2.58) remained independent predictors of overweight and obesity.

Conclusions

More than one-fifth of adolescents in this urban Chennai school were overweight or obese, highlighting a substantial and growing public health concern. Modifiable lifestyle factors, particularly screen exposure and fast-food consumption, play a significant role. Strengthening school-based and family-centered interventions focusing on healthy eating habits, physical activity, and reduced sedentary behavior is essential to address adolescent overweight and obesity and support long-term health and well-being.

## Introduction

Over the past few decades, the global prevalence of childhood and adolescent obesity has risen dramatically, representing one of the most serious public health challenges of the 21st century [1]. As per the World Health Organisation (WHO), it is estimated that over 390 million children and adolescents aged 5-19 years were overweight or obese in 2022 [2]. Obesity, once a problem in high-income nations, is rapidly increasing in middle-income countries and low-income countries, more particularly in urban areas of Asia.

In India, the nutritional transition toward energy-dense diets, combined with minimal physical activity, has led to a dual burden of malnutrition. While undernutrition persists, childhood overweight and obesity are steadily increasing, especially in metropolitan cities like Chennai [3]. Rapid urbanisation, easy access to fast food, sedentary recreational habits, and increased academic pressure have all contributed to this trend.

Adolescence is considered to be a critical period with marked rapid growth and change in behaviour. Excess weight during this phase increases the chance of adult obesity and associated non-communicable diseases such as type 2 diabetes, hypertension, and cardiovascular disease. Early detection and intervention during adolescence are therefore crucial.

Although obesity in teenagers has been studied in many parts of India, data from major southern metropolitan cities, particularly Chennai, remain limited. Understanding local lifestyle factors like diet, sleep, and screen use will help in planning interventions that fit the community.

This study was done to determine the prevalence of overweight and obesity among adolescent school children in Chennai city and to identify associated sociodemographic and behavioural risk factors.

## Materials and methods

Study design and setting

A school-based cross-sectional study was conducted among adolescent school children in an urban area of Chennai, Tamil Nadu, India. The study period was from January to April 2025.

Study population and sampling

The study was conducted in one higher secondary school among students studying in classes IX, X, XI, and XII. These classes were included based on administrative feasibility and permission granted by the school authorities. Thereby, the study population consisted of adolescents aged 13-18 years. All students who were present on the day of data collection and who provided assent, along with written informed consent from parents or guardians, were included in the study. Students who were absent on the day of data collection or unwilling to participate were excluded.

Sample size calculation

The sample size was calculated based on a previously reported prevalence of overweight and obesity of 14.7% among adolescents by Kotian et al. [4]. Using an absolute precision of 5% and a confidence level of 95%, the minimum required sample size was calculated using the formula: n = (Z² × p × q) / d². Therefore, the calculated sample size was 193. However, a total of 222 adolescents participated in the study.

Study tool and variables

Data were collected using a pre-tested, semi-structured questionnaire developed by the investigators. The questionnaire included sections on socio-demographic characteristics (age, sex, religion, and socioeconomic status), dietary habits (frequency of fast-food consumption and meal timing), and lifestyle behaviours (physical activity, sleep habits, and daily screen time). The questionnaire was pre-tested among adolescents from a different school, and necessary modifications were made to ensure clarity and relevance. Socioeconomic status was assessed using the modified BG Prasad classification (2025 update), based on per capita income, which is available in the public domain [5].

Operational definitions

Excess screen time was defined as more than two hours per day. Frequent fast-food intake was defined as consumption of fast food three or more times per week. Late dinner was defined as consumption of the main evening meal after 9:00 PM. Adequate sleep was defined as a sleep duration of eight hours or more per day.

Anthropometric measurements

Anthropometric measurements were obtained using standardized procedures. Height was measured to the nearest 0.1 cm using a stadiometer with participants standing erect without footwear. Weight was measured to the nearest 0.1 kg using a calibrated digital weighing scale. Body mass index (BMI) was calculated as weight in kilograms divided by height in meters squared (kg/m²). Nutritional status was assessed using BMI-for-age Z-scores based on the WHO growth reference for children and adolescents aged 5-19 years, which are publicly accessible from the WHO growth reference data website [6]. Overweight was defined as BMI-for-age > +1 standard deviation (SD), and obesity as > +2 SD.

Ethical considerations

Ethical approval was obtained from the Institutional Ethics Committee of Sri Ramachandra Institute of Higher Education and Research before the commencement of the study (REF NO: CSP-MED/24/AUG/107/261 dated 09.06.2024). Written informed consent was obtained from parents or guardians, and assent was obtained from all participating adolescents. Confidentiality and anonymity of participants were strictly maintained.

Statistical analysis

Data were entered into Microsoft Excel and analysed using IBM SPSS Statistics for Windows, Version 29 (Released 2023; IBM Corp., Armonk, New York, United States). Descriptive statistics were used to summarise participant characteristics. Associations between overweight/obesity and independent variables were assessed using the chi-square test and odds ratios with 95% confidence intervals. Variables that were statistically significant in bivariate analysis were entered into a multivariate logistic regression model to identify independent predictors of overweight and obesity. A p-value < 0.05 was considered statistically significant.

## Results

A total of 222 adolescents were included in the study. Among them, 201 (90.5%) were boys, and 21 (9.5%) were girls. The mean age of the adolescents was 14.99 ± 1.18 years, with a higher proportion belonging to the late adolescent age group (≥15 years) of 57.65%. Most participants belonged to the Hindu religion (90.1%) and were from upper socio-economic (88.7%) strata (Table 1).

**Table 1:** Background characteristics of the study participants (n=222) Socio-economic status was classified using the modified BG Prasad scale (2025 update) [5].

S.No	Factors		Frequency	Percentage
1	Sex	Male	201	90.5 %
		Female	21	9.5%
2	Age	Early Adolescents	94	42.3 %
		Late Adolescents	128	57.7 %
3	Religion	Hindu	200	90.1 %
		Muslim	10	4.5 %
		Christian	12	5.4 %
4	Socio-Economic Status	Upper Class	197	88.7 %
		Upper Middle	22	9.9 %
		Lower Middle	2	0.9 %
		Upper Lower	0	0 %
		Lower	1	0.5 %

Based on the BMI for age Z scores as per the WHO growth reference for children and adolescents aged 5-19, 7.7% of adolescents were overweight, 14 % were obese. Overall, the study shows that 21.7% of participants are overweight or obese (Table 2).

**Table 2:** Prevalence of overweight and obesity (n=222) BMI classification based on WHO growth reference for children and adolescents aged 5–19 years [6].

Factors		Frequency	Percentage
BMI	Severe Thinness	19	8.6 %
	Thinness	73	32.9 %
	Normal BMI	82	36.9 %
	Overweight	17	7.7 %
	Obesity	31	14 %

Female adolescents showed a higher prevalence of overweight and obesity compared to male adolescents (OR = 6.11; 95% CI: 2.40-15.60; p < 0.001), which is statistically significant. Adolescents reporting screen time exceeding two hours per day had more than twice the likelihood of being overweight or obese (OR = 2.26; p = 0.021). Similarly, frequent consumption of fast food (OR = 2.49; p = 0.006) and having dinner after 9 PM (OR = 2.12; p = 0.038) also showed an association with excess body weight, which is statistically significant. No statistically significant association was observed with sleep duration and snoring (Table 3).

**Table 3:** Association of selected risk factors with overweight and obesity among adolescents (n=222) * p-value < 0.05 is significant Late adolescent age group: ≥15 - 18 years; early adolescent age group: 13–14 years; frequent fast-food intake: ≥3 times per week

S. No	Risk Factors		Obesity and Overweight		Odds Ratio	95 % CI	p-Value
			Yes (n-48)	No (n-174)			
1	Gender	Female	12	9	6.111	2.40-15.60	<0.001*
		Male	36	165			
2	Age	Early Adolescents	18	76	0.774	0.40-1.49	0.443
		Late Adolescents	30	98			
3	Screen Time Per Day	>2 hours	17	34	2.26	1.12 –4.55	0.021*
		<2 hours	31	140			
4	Dinner Time	Before 9 PM	36	102	2.12	1.03 - 4.35	0.038*
		After 9 PM	12	72			
5	Frequent Fast-Food Intake	Yes	23	47	2.486	1.29-4.80	0.006*
		No	25	127			
6	Sleep Time	>8 hrs	33	123	0.912	0.46-1.82	0.795
		<8 hrs	15	51			
7	Snoring During Sleep	Yes	6	40	0.48	0.19-1.20	0.112
		No	42	134			

On multivariate logistic regression analysis, female gender (AOR = 6.52; p < 0.001), increased daily screen exposure (AOR = 2.46; p = 0.017), and frequent fast-food intake (AOR = 2.58; p = 0.017) emerged as independent predictors of overweight and obesity, while the association with late dinner timing lost statistical significance after adjustment (Table 4).

**Table 4:** Multivariate logistic regression analysis of factors associated with overweight and obesity (n=222) * p-value < 0.05 is significant Frequent fast-food intake (≥3 times per week)

S.no	Variables		Unadjusted Odds ratio (95% CI)	p-value	Adjusted Odds ratio (95% CI)	p-value
1	Gender	Female	6.11 (2.40-15.60)	<0.001	6.52 (2.45-17.36)	<0.001*
		Male	1 (ref)			
2	Screen Time Per Day	>2 hours	2.26 (1.12-4.55)	0.021	2.46 (1.18-5.29)	0.017*
		<2 hours	1 (ref)			
3	Dinner Time	After 9 pm	2.12 (1.03-4.35)	0.038	0.95 (0.47-1.95)	0.895
		Before 9 pm	1 (ref)			
4	Frequent Fast-Food Intake	Yes	2.49 (1.29-4.80)	0.006	2.58 (1.28-5.19)	0.017*
		No	1 (ref)			

## Discussion

The present study shows that nearly one in five adolescents in a selected urban higher secondary school in Chennai is either overweight or obese, indicating a growing nutritional and lifestyle burden in urban settings. The observed prevalence of 21.7% is consistent with reports from other major Indian metropolitan cities, where adolescent overweight and obesity rates have been documented between 15% and 25% [3,7,8]. Before the COVID-19 pandemic, NFHS-4 (2015-16) data revealed a combined prevalence of obesity and overweight of 8% among adolescents (15-19 years) in Tamil Nadu. The Comprehensive National Nutrition Survey (CNNS 2016-18) documented a combined prevalence of overweight and obesity of 12-15% among adolescents in Tamil Nadu [9]. In contrast, this study demonstrates a disproportionately higher prevalence of overweight and obesity, exceeding pre-pandemic values. This difference may be attributed to sedentary lifestyles, frequent consumption of energy-dense foods, prolonged screen time, and reduced physical activity. Additionally, the higher obesity prevalence compared to overweight observed in this study shows a shift from early excess weight to more established obesity, suggesting delayed identification and limited early preventive interventions. The prevalence observed in this study is similar to trends in other developing Asian nations, such as Malaysia (22%) [10] and the Philippines (19%) [11]. However, this prevalence is lower than that reported in high-income countries such as the United States (42.5%) [12]. This similarity suggests that urban environments across India share comparable dietary patterns, lifestyle behaviours, and exposure to factors influencing obesity.

Female adolescents in this study demonstrated a significantly higher risk of overweight and obesity, with odds exceeding six times that of male adolescents. The lower proportion of female participants reflects the actual enrolment pattern in the selected school, as all eligible students were included in the study. This gender variation is well supported in the literature and may be justified by several socio-behavioural factors. Girls often engage in lower levels of physical activity due to cultural restrictions on outdoor play, safety concerns, household responsibilities, and reduced participation in organised sports [13,14]. Additionally, psychosocial factors may influence eating behaviours and weight gain among adolescent girls. Comparable gender differences have been reported in studies from Kerala and Karnataka, suggesting that this is a widespread pattern across Indian urban populations [4,15].

Frequent consumption of fast food (≥3 times per week) remained a significant determinant of overweight even after adjusting for confounders. This finding is biologically and behaviourally justified, as fast foods are typically energy-dense, high in saturated fats, added sugars, and refined carbohydrates. Easy accessibility to such foods and aggressive marketing directed at children and adolescents amplify their intake [16]. Similar associations have been reported in Lucknow, Uttar Pradesh, and Mangalore [17,18], indicating that fast-food consumption is a major modifiable risk factor across the country.

Late-night dinners (after 9 PM) showed a significant association in bivariate analysis. This relationship is supported by studies demonstrating that late-night eating has been associated with circadian disruption, reduced insulin sensitivity, and greater postprandial fat storage [19,20].

Excessive screen exposure (>2 hours/day) emerged as an important predictor, doubling the risk of overweight. This finding aligns with established evidence that prolonged screen time promotes sedentary behaviour, reduces time available for physical activity, and encourages passive snacking [21,22]. Screen exposure has been associated with impaired sleep quality and increased night-time alertness due to blue-light emission, indirectly contributing to weight gain [23].

There was no significant relationship between being overweight and sleep duration. Similar findings have been reported in studies where sleep influenced weight only in cases of chronic or marked sleep deprivation [24]. Additionally, the homogeneity of socio-economic status, predominantly upper class, limited its variability and thereby its influence on outcomes.

On performing multivariate regression analysis of our study results, the female gender had a stronger predilection for obesity compared to the male gender. Similarly, the association of excessive screen exposure and frequent fast- food intake with obesity stands still more significant. Loss of significance between dinner time and obesity was observed, suggesting that the effect may be mediated by overall dietary patterns or energy intake, rather than dinner timing alone.

Overall, the rising burden of adolescent obesity in India suggests an integration between the global urban dietary habits and lifestyle changes. The dual impact of westernised food habits and increasingly sedentary routines across urban Asia highlights the urgency for culturally relevant, region-specific overweight and obesity prevention programs among adolescents.

Strengths

This study provides important data on adolescent overweight and obesity from Chennai, where such information is limited. It has the strength of using accurately measured height and weight instead of self-reports, reducing bias. By assessing key behaviours such as diet, screen time, and sleep patterns, the study offers a well-rounded view of modifiable lifestyle factors, increasing the practical usefulness of the findings. The use of logistic regression adds analytical strength by adjusting for confounders and identifying independent risk factors.

Limitations

As the study was conducted in a single school using convenience sampling, the findings may not be generalisable to all adolescents in Chennai or Tamil Nadu. Gender imbalance, particularly the lower number of female participants, may affect gender-based comparisons. Social desirability bias is possible due to self-reported data on lifestyle and behavioural habits. 

## Conclusions

Overweight and obesity affect more than one out of five adolescents in Chennai, signalling an alarming public health challenge in urban India. Girls and those who frequently consume fast food are particularly at risk, showing how gender, lifestyle, and cultural factors combine to influence health in adolescents. These findings show the urgent need for proactive measures and behavioural change communication, especially in schools, where children spend more time and where health habits can be shaped early. Interventions like nutrition education, counselling on healthy food choices, and promotion of physical activity can make a meaningful difference. Beyond schools, involving families and communities is essential to create environments that support healthier lifestyles, reduce sedentary behaviour, and encourage balanced diets. Addressing adolescent overweight and obesity is not just about numbers or statistics; it underscores the importance of equipping adolescents with knowledge and skills to adopt healthier lifestyles and prevent long-term complications like diabetes, cardiovascular disease, and reduced quality of life. Early, culturally sensitive, and sustainable interventions can help adolescents make informed choices, develop resilience against unhealthy lifestyle pressures, and carry these positive habits into adulthood. By acting now, we have the opportunity to turn the tide on a rising epidemic and empower the next generation to thrive both physically and mentally.

## Appendices

Questionnaire

https://docs.google.com/document/d/1EquvGJR_1EOoqRW1fMzBKoFcHi17stDk/edit?usp=sharing&ouid=116714201660768138355&rtpof=true&sd=true
